# Numerical Study of Graphene/Au/SiC Waveguide-Based Surface Plasmon Resonance Sensor

**DOI:** 10.3390/bios11110455

**Published:** 2021-11-15

**Authors:** Wei Du, Lucas Miller, Feng Zhao

**Affiliations:** 1 Department of Electrical Engineering and Physics, Wilkes University, Wilkes-Barre, PA 18766, USA; lucas.miller@wilkes.edu; 2 Micro/Nanoelectronics and Energy Laboratory, School of Engineering and Computer Science, Washington State University, Vancouver, WA 98686, USA

**Keywords:** waveguide, surface plasmon resonance, graphene, silicon carbide, refractive index, chemical sensor, biosensor

## Abstract

A new waveguide-based surface plasmon resonance (SPR) sensor was proposed and investigated by numerical simulation. The sensor consists of a graphene cover layer, a gold (Au) thin film, and a silicon carbide (SiC) waveguide layer on a silicon dioxide/silicon (SiO_2_/Si) substrate. The large bandgap energy of SiC allows the sensor to operate in the visible and near-infrared wavelength ranges, which effectively reduces the light absorption in water to improve the sensitivity. The sensor was characterized by comparing the shift of the resonance wavelength peak with change of the refractive index (RI), which mimics the change of analyte concentration in the sensing medium. The study showed that in the RI range of 1.33~1.36, the sensitivity was improved when the graphene layers were increased. With 10 graphene layers, a sensitivity of 2810 nm/RIU (refractive index unit) was achieved, corresponding to a 39.1% improvement in sensitivity compared to the Au/SiC sensor without graphene. These results demonstrate that the graphene/Au/SiC waveguide SPR sensor has a promising use in portable biosensors for chemical and biological sensing applications, such as detection of water contaminations (RI = 1.33~1.34), hepatitis B virus (HBV), and glucose (RI = 1.34~1.35), and plasma and white blood cells (RI = 1.35~1.36) for human health and disease diagnosis.

## 1. Introduction

Surface plasmons resonance (SPR)-based chemical and biosensors have been widely used for the detection of a variety of chemicals and biomolecules [1,2,3,4,5]. These sensors utilize the surface plasmon polariton (SPP) characteristics to analyze the sensing medium. Change of the analyte concentration on the sensor surface leads to change of local refractive index (RI), and therefore the propagation constant of SPP, which can be measured by optical methods [6]. SPR sensors can be built with several configurations. The most frequently used structures are a prism coupler [7] and a grating coupler [8], in which the resonance angle shifts with the change of RI. However, limitations exist in both sensors, as the prism structure suffers from a relatively large device size, while the grating structure has a small size but complicated fabrication process. Both configurations are not conducive to future photonics integration needs. To address these issues, the optical waveguide-based structures are attracting interest [9,10,11] since they offer the advantages of good control of light path, easy measurement of input and output light intensity, and a high degree of on-chip integration. These advantages are highly desirable for the next generation of photonic integrated circuits (PICs) [12].

In conventional SPR sensors, a thin metal film is inserted between the light-guiding layer and sensing medium, with the excited surface plasmon wave propagating along the metal and sensing medium interface. Different metal films such as gold (Au), silver (Ag), and aluminum (Al) have been discussed in the literature [13,14,15]. Among these metals, Au is widely used due to its resistance to chemicals and oxidation. However, the intrinsic high loss of Au film limits the sensitivity. Various methods [3] have been developed to further improve the sensitivity, including the application of metal nanoparticles [16] and nanoslits [17]. Since the precise control of nanostructures is a challenge, these methods complicate the sensor fabrication toward on-chip integration and are not compatible with a silicon (Si) complementary metal–oxide–semiconductor (CMOS) technique. An alternative approach is the application of biomolecular recognition elements (BRE) [3] to functionalize the Au film and enhance the adsorption of analyte on the Au surface.

In this paper, we proposed and numerically simulated a new waveguide-based SPR sensor with a wide bandgap semiconductor silicon carbide (SiC) as the waveguide layer, a Au thin film, and a graphene cover layer as BRE. Due to the wide bandgap energy (2.2 eV of 3C-SiC polytype), the SiC waveguide layer enables the sensor to operate in the visible wavelength range, which avoids the absorption of large amounts of water in the near-infrared range when sensing in a water-based sensing medium [18,19,20]. The increased adsorption of the analyte on a graphene surface could improve the sensitivity of the sensor [21,22]. The graphene/Au/SiC waveguide SPR sensor structure can be grown and fabricated using standard Si CMOS techniques: SiC can be grown using plasma—enhanced chemical vapor deposition (CVD) [23] on SiO_2_/Si substrate, Au can be deposited via evaporation or sputtering with precisely controlled thickness. For graphene, CVD process has been explored extensively to synthesize uniform graphene monolayer and multilayer [24], which can be used to accurately deposit on the SiC surface. Furthermore, the standard photolithography and dry etching processes can be applied to pattern the device.

## 2. Design and Methods

**Figure 1:**
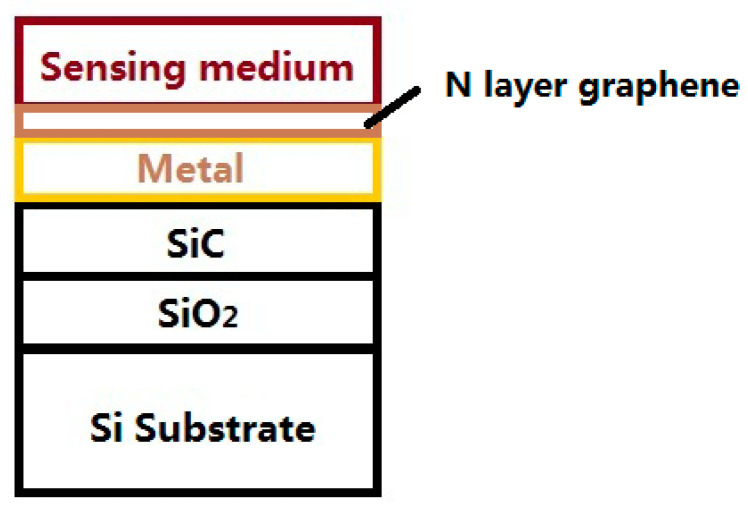
Cross-sectional schematic of graphene/Au/SiC waveguide SPR sensor structure.

The cross-sectional schematic of the graphene/Au/SiC waveguide SPR sensor is shown in Figure 1. The width of the sensor was kept at 10 µm, which is attainable via the standard microfabrication process. A SiO_2_ layer (RI = 1.45) was inserted between a Si (RI = 3.5) substrate and SiC waveguide layer for optical isolation since the RI of SiC (RI = 2.62) is smaller than Si. The permittivity of Au can be found in [25]. The SiO_2_ isolation layer was selected to be 3 μm thick, which is sufficient to prevent optical field leakage onto the Si substrate. Thicknesses of the SiC waveguide layer was kept at 100 nm based on our previous study [26], which showed that the confinement factor of a SiC waveguide sensor increases with the thickness of the SiC layer up to 100 nm. Above this thickness, the coupling effect becomes weak, which reduces the coupling coefficient and confinement factor. Thicknesses of the Au film (from 10 to 100 nm) and graphene layer were investigated in this study. The thickness of the graphene monolayer was 0.34 nm [27] so the total thickness of graphene was N × 0.34 nm, where N varied from 0 (no graphene) to 10 in this study. The sensor was studied by a spectral interrogation method; the resonance wavelength was collected, and its shift with the change in the RI of the sensing medium being compared. This method is different from the angular interrogation method in prism-or grating-based SPR sensors, which use the resonance angle instead of the resonance wavelength.

The optical properties of a monolayer graphene can be found in [27], which provides the complex RI of graphene in the visible light range:

(1) $$RI_{\left(graphene\right)}=3.0+i\frac{5.446\;{\mu m}^{-1}}{3}\lambda_{0}$$

where *i* is the imaginary unit and *λ*_0_ is the vacuum wavelength. In [27], the optical properties were reported for single and multiple graphene layers and the quantum effects studied. Therefore, application of Equation (1) for the RI of graphene includes quantum effects that could present the multi-layer of graphene. For sensor operation, when the resonance condition is satisfied by a certain RI, the SPP is excited by the incident light, leading to the optical energy distribution in both the waveguide layer and sensing medium. The optical energy being absorbed by the analyte in the sensing medium results in attenuation of the output power. A change of analyte concentration alters the RI near the sensor surface, which consequently changes the propagation constant of SPP and eventually leads to a shift in the resonance wavelength. According to Lambert–Beer’s law, in a waveguide structure, the absorbance, *A*, can be expressed as [28,29]:

(2) $$A=\log\left(\frac{P_{0}}{P_{a}}\right)=f\alpha lc$$

where *P*_0_ and *P_a_* are the light intensities without and with energy absorption in the sensing medium, respectively; *f* is the confinement factor, which is defined as the ratio of the optical power confined in the sensing medium over the total optical power; *α* is the absorption coefficient; *l* is the waveguide length; and *c* is the analyte concentration. The change in *c* leads to the change in RI. The sensitivity, *S*, is proportional to the ratio of the change in absorbance, *A*, over the concentration change in the sensing medium:

(3) $$S=\frac{\Delta A}{\Delta c}=f\alpha l$$

It is clear in Equation (3) that for a given waveguide length, a higher confinement factor value results in an improved sensitivity of the waveguide sensor. In addition, with the graphene layer serving as BRE, the adsorption efficiency is enhanced. This can be defined as the percentage of analyte being effectively adsorbed into the sensor surface, which leads to the change of RI. Therefore, the overall sensitivity, *S*, is improved by adding graphene layers, as shown in Equation (4):

(4) $$S\propto k\cdot \frac{\Delta A}{\Delta c}=kf\alpha l$$

where *k* is the enhancement of adsorption efficiency and *k* > 1.

Note that in Equation (4), the values of *k* and *α* are generally obtained by experimental measurement. In this study, we applied an effective index method (EIM) [30,31] using COMSOL Multiphysics software to model the graphene/Au/SiC waveguide SPR sensor and calculate the confinement factor *f*. The results were compared with the Au/SiC waveguide SPR sensor without a graphene layer. In the model, a 3-μm thick perfectly matched layer (PML) surrounding the sensor structure was used as the outer boundary condition to truncate the computation region. At the boundary of each layer, the tangential components of the electric and magnetic fields were defined as the continuous inner boundary condition. Since SPP can only be excited by transverse magnetic (TM) polarized light, and the fundamental TM mode (TM_0_) in the waveguide has the lowest loss, the TM_0_ mode was investigated.

## 3. Results and Discussion

**Figure 2:**
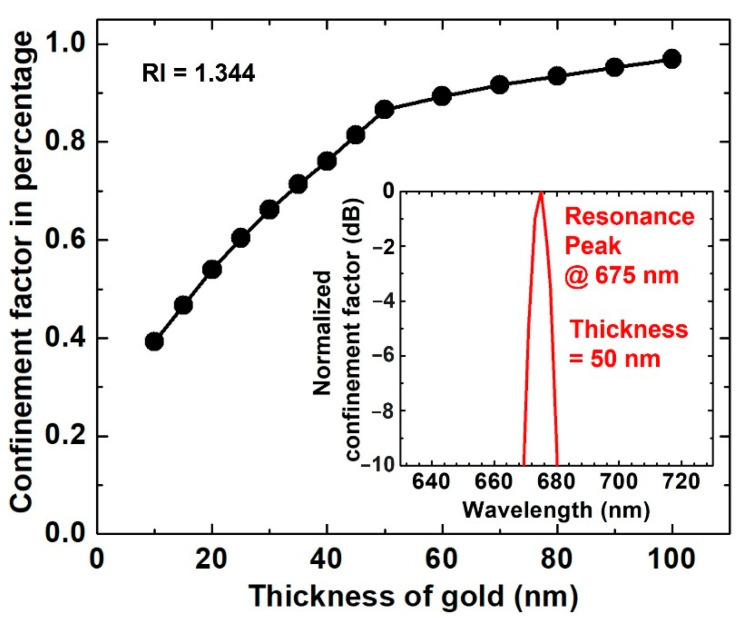
Confinement factor as a function of Au thickness. The number of graphene layers was 10. The RI of the sensing medium was 1.344. Inset: resonant peak at 675 nm with 50 nm thick gold.

Figure 2 shows the confinement factor of the TM_0_ mode as a function of Au film thickness at its resonance wavelength (675 nm, see inset) in a sensing medium where RI = 1.344. Ten graphene layers were applied to the Au film. The confinement factor increases as the Au film becomes thicker. Considering that thicker Au would lead to higher optical loss, the 50 nm thickness was selected in this sensitivity study of the graphene/Au/SiC waveguide SPR sensor. This thickness allowed for strong coupling between the waveguide and SP modes.

**Figure 3:**
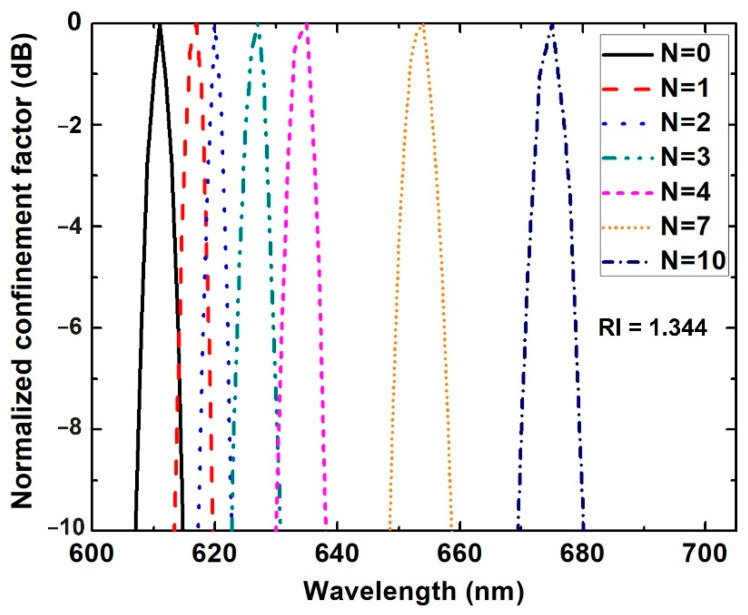
Resonance wavelength for graphene/Au/SiC sensor with different numbers of graphene layers. The gold thickness was 50 nm. The RI of the sensing medium was 1.344.

The typical resonance wavelength shift for different numbers of graphene layers in a RI = 1.344 sensing medium is shown in Figure 3. It is clear that, as the number of graphene layers increases, the resonance wavelength shifts towards longer wavelengths, from 617 to 675 nm, i.e., a redshift. It is well acknowledged that the resonance wavelength (or frequency) is strongly dependent on the free-electron density of metallic nanostructures [32]. Without the graphene layer, the resonance wavelength is mainly determined by the excited electron density in the Au thin film. When graphene is directly added to the Au film, the electrons excited by SPP can transfer to the graphene, resulting in decreased free-electron density in the Au film. This reduced electron density leads to the redshift of the resonance wavelength. As graphene layers increase, more electrons transfer to the graphene layers and free-electron density further decreases, therefore a more pronounced redshift of the resonance peak is observed. It is worth noting that as the number of graphene layers increases, the linewidth broadens slightly, which may degrade sensing performance. A similar characteristic was also observed in a prism-coupler-based SPR sensor [22]. However, since adding more graphene layers would cause a further shift of the resonance wavelength as shown in Figure 3, this effect is compensated for and the overall sensitivity can still be improved.

**Figure 4:**
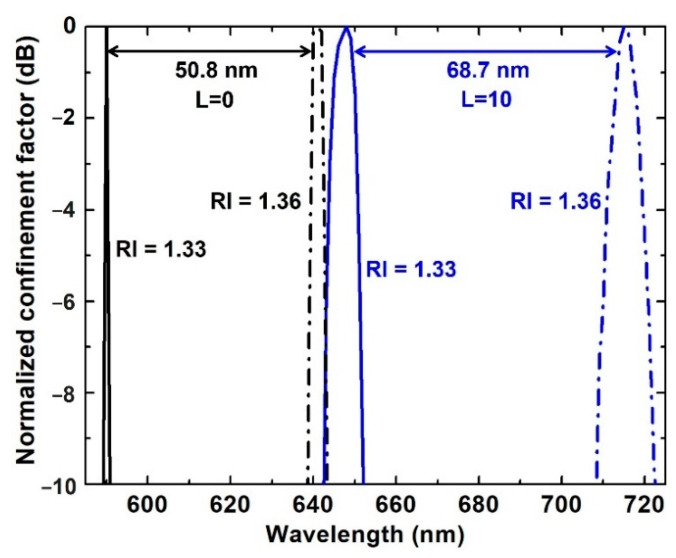
Comparison of resonance wavelength shift for a Au/SiC (black curves, N = 0) and a graphene/Au/SiC (blue curves, N = 10) waveguide-based SPR sensor when the RI of the sensing medium changes from 1.33 (solid curves) to 1.36 (dashed curves).

Modification of the SPP field also improves sensitivity. Figure 4 shows the resonance-wavelength shift of the graphene/Au/SiC waveguide SPR sensor with 10 layers of graphene (blue curves, N = 10) in comparison with the Au/SiC sensor (black curves, N = 0) without graphene; the two RIs mimic the change of analyte concentration. As the RI of the sensing medium increases from 1.33 to 1.36, the resonance wavelength of both sensors shifts to the longer wavelength. The resonance wavelength peak indicates the strongest SPR coupling from waveguide mode to surface plasmon (SP) mode, where the maximum propagation loss of the waveguide occurs. In SP mode, the maximum propagation loss of the waveguide is close to the cutoff wavelength. The increase in RI leads to a shift of the cutoff wavelength towards a longer wavelength and therefore the redshift of the resonance wavelength peak. The graphene/Au/SiC sensor exhibits a redshift of 68.7 nm, corresponding to a sensitivity of 2290 nm/RIU (refractive index unit). While for the Au/SiC sensor, the resonance wavelength shifts 50.8 nm, i.e., a sensitivity of 1693 nm/RIU. An improvement of 35.8% was obtained by adding 10 graphene layers to the Au/SiC waveguide SPR sensor.

**Figure 5:**
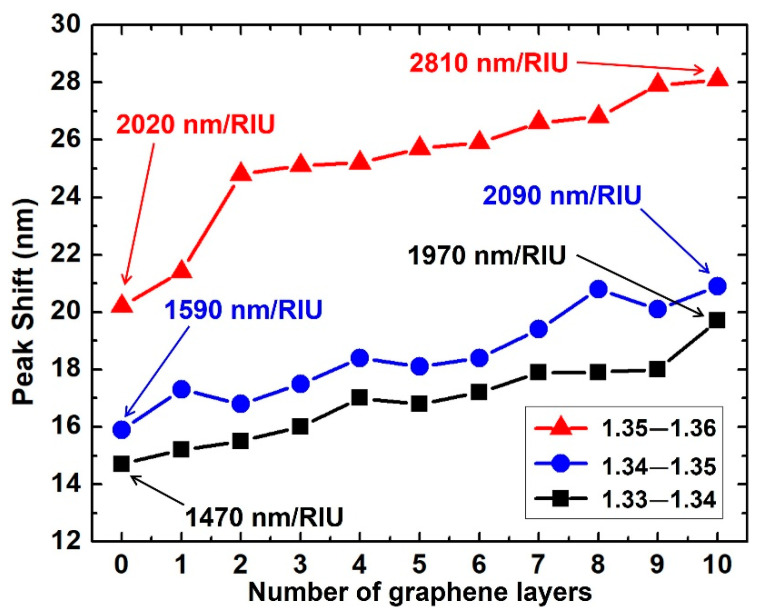
Resonance peak shift with the number of graphene layers. The gold thickness was 50 nm and RI ranges from 1.33 to 1.36.

The sensitivities in the three RI ranges (1.33~1.34, 1.34~1.35, and 1.35~1.36) are summarized in Figure 5. In each RI range, an increased sensitivity is demonstrated with more graphene layers. For 10 graphene layers, the sensitivities of 1970 nm/RIU, 2090 nm/RIU and 2810 nm/RIU were obtained in each RI range, corresponding to the enhancements of 34.0%, 31.4%, and 39.1%, respectively. There are very useful sensing applications when a graphene/Au/SiC waveguide SPR sensor is used in these RI ranges: detection of contaminations in water [33] in RI = 1.33~1.34, and identification of hepatitis B virus (HBV) [34], glucose [35] in RI = 1.34~1.35, and plasma and white blood cells in RI = 1.35~1.36 [36]. Detection of refractive index change is a useful technique for biosensors and continuously attracting research interest [37,38,39]. Very small changes in concentration can be detected by utilizing the sensitivity that has been improved by SPR effect.

It is worth noting that according to Equation (4), both the absorption coefficient, *α*, and the enhanced adsorption efficiency, *k*, are dependent on analyte materials; therefore, the sensitivity of the waveguide SPR sensor would vary when probing different analytes. Moreover, more sensitivity improvement can be expected since k is always greater than one when adding more graphene layers. However, further coverage of additional graphene layers may not effectively further the transfer of the electrons from the Au film to the graphene. As a result, the resonance peak shift would reach its maximum and no more peak shift could be obtained. On the other hand, the broadened peak linewidth with increased graphene layers also needs to be considered, and the optimal number of graphene layers for maximal sensitivity requires future study.

## 4. Conclusions

In this work, we proposed and investigated a new graphene/Au/SiC waveguide SPR sensor. The confinement factors of the sensors with 0 to 10 graphene layers were studied by an effective index method, and the sensitivity was studied by a spectral interrogation method by comparing the shift in the resonance wavelength peak. The sensor operates in the visible light range with the wide bandgap SiC as the waveguide layer. When combined with the advantages of good control of light path, easy measurement of input and output light intensity, and high degree of on-chip integration, this sensor is highly desirable for the next generation of PICs. This study shows that when the number of graphene layers is increased, sensitivity increases, and up to 2810 nm/RIU can be achieved in the RI range of 1.33~1.36. The results demonstrate that a graphene/Au/SiC waveguide SPR sensor is promising for important chemical and biological sensing applications.

## Data Availability

The data presented in this study are available on request from the corresponding author.
